# Recent advances in research on phosphate starvation signaling in plants

**DOI:** 10.1007/s10265-024-01545-0

**Published:** 2024-04-26

**Authors:** María Isabel Puga, César Poza-Carrión, Iris Martinez-Hevia, Laura Perez-Liens, Javier Paz-Ares

**Affiliations:** Department of Plant Molecular Genetics, Centro Nacional de Biotecnologia-CSIC Campus Universidad Autonoma, Darwin 3, Madrid, 28049 Spain

**Keywords:** Arabidopsis, Crosstalk, Mycorrhiza, Microbiome, Natural variation, Nutrient, Phosphate starvation signaling, Phosphorus, Plant, Rice

## Abstract

Phosphorus is indispensable for plant growth and development, with its status crucial for determining crop productivity. Plants have evolved various biochemical, morphological, and developmental responses to thrive under conditions of low P availability, as inorganic phosphate (Pi), the primary form of P uptake, is often insoluble in soils. Over the past 25 years, extensive research has focused on understanding these responses, collectively forming the Pi starvation response system. This effort has not only expanded our knowledge of strategies to cope with Pi starvation (PS) but also confirmed their adaptive significance. Moreover, it has identified and characterized numerous components of the intricate regulatory network governing P homeostasis. This review emphasizes recent advances in PS signaling, particularly highlighting the physiological importance of local PS signaling in inhibiting primary root growth and uncovering the role of TORC1 signaling in this process. Additionally, advancements in understanding shoot-root Pi allocation and a novel technique for studying Pi distribution in plants are discussed. Furthermore, emerging data on the regulation of plant-microorganism interactions by the PS regulatory system, crosstalk between the signaling pathways of phosphate starvation, phytohormones and immunity, and recent studies on natural variation in Pi homeostasis are addressed.

## Introduction

Since the advent of molecular genetics, the phosphate starvation response system (PSR), initially in microorganisms and later also in plants has served as emblematic systems for studying gene regulation. Recently, in plants there has been a growing interest in these studies due to their potential to inform strategies aimed at reducing the reliance on phosphate fertilizers, a crucial step towards fostering effective sustainable agricultural practices. These responses not only enhance phosphate acquisition and utilization efficiency but also shield plants from the stress induced by Pi starvation (reviewed by Lopez-Arredondo et al. 2014; Ojeda-Rivera et al. 2022; Paz-Ares et al. 2022).

Among the responses to phosphate starvation (PS) aimed at increasing Pi acquisition observed across a broad range of species are the increase of phosphate transporter activity, phosphatases, RNAses, and proton and organic acid secretion, which result in improved Pi mobilization, acquisition (Bariola et al. 1994; Plaxton and Tran 2011; Raghothama 1999; Taylor et al. 1993). In addition, PS triggers lipid remodeling, thereby reducing internal Pi requirements (Nakamura 2013; Okazaki et al. 2013), and a reduction in shoot growth and branching, alongside remodeling of root system architecture involving increased lateral roots and root hair length and number, and a reduction in primary root growth. This restructuring facilitates the development of a shallower root system efficient at exploring topsoil resources rich in available Pi, particularly in non-fertilized lands (Lopez-Bucio et al. 2003; Lynch 2011; Lynch and Brown 2001). PS also induces responses that promote beneficial plant-microbe interactions, exemplified primarily by symbiosis with mycorrhizae but extending beyond these fungal species (Paries and Gutjahr 2023; Smith and Smith 2011; Zhao et al. 2023). Furthermore, some species develop proteoid roots, clusters of lateral roots that secrete protons in small volumes to efficiently extract Pi (Lambers et al. 2015; Neumann and Martinoia 2002). A recent addition to the arsenal of the PSR is the induction of carnivorous leaves, as observed in the African plant *Triphyophyllum peltatum* (Winkelmann et al. 2023). The role of carnivory in nutrient acquisition has been known for long time (reviewed by Adamec 1997; Ellison 2006). The discovery of conditional PS-dependent carnivory in *T. peltatum* particularly underscores the functional association between carnivorous behavior and phosphorus (P) nutrition. It will be interesting to dissect the molecular details underlying recruitment of carnivory under the PS response regulatory system.

Underlying the PSR is a sophisticated and complex regulatory system extensively studied since the early 21st century when the first regulator, PHR1 transcription factor (TF), was identified in Arabidopsis (Rubio et al. 2001). Presently, many details of this regulatory system are known; it involves two partially independent signaling pathways one responding to local soil Pi levels and another responding to intracellular Pi concentrations which can operate systemically at long distance (Burleigh and Harrison 1999; Thibaud et al. 2010). Key components include the STOP1 transcription factor and LPR1-like ferroxidases for local signaling, and PHR1(-like) TF and SPX sensors for systemic signaling. Over 20 signaling components, including TFs, SPX sensors, kinases, phosphatases, ferroxidases, miRNAs, and miRNA inhibitors, have been identified, collectively orchestrating Pi homeostasis. This review focuses on recent discoveries validating the physiological relevance of local PS signaling and revealing the implication of TORC1 signaling in regulating root growth under low Pi conditions. Additionally, it highlights recent advances in understanding shoot-root Pi allocation and refers to a new technical breakthrough enabling histochemical studies of intracellular Pi distribution in planta. Finally, it touches upon new information on the control of arbuscular mycorrhizal symbiosis by PHR1, the implication of FERONIA (FER) and PHR1 in reducing pathogen defenses under low Pi, which help in shaping the PS microbiome, and the reciprocal implication of plant immunity signaling in decreasing Pi uptake, alongside the crosstalk between signaling pathways of Pi starvation and phytohormones, as well as recent research exploring and exploiting natural variation in Pi homeostasis.

### The control of growth under pi deficiency: local PS signaling and beyond

**Fig. 1:**
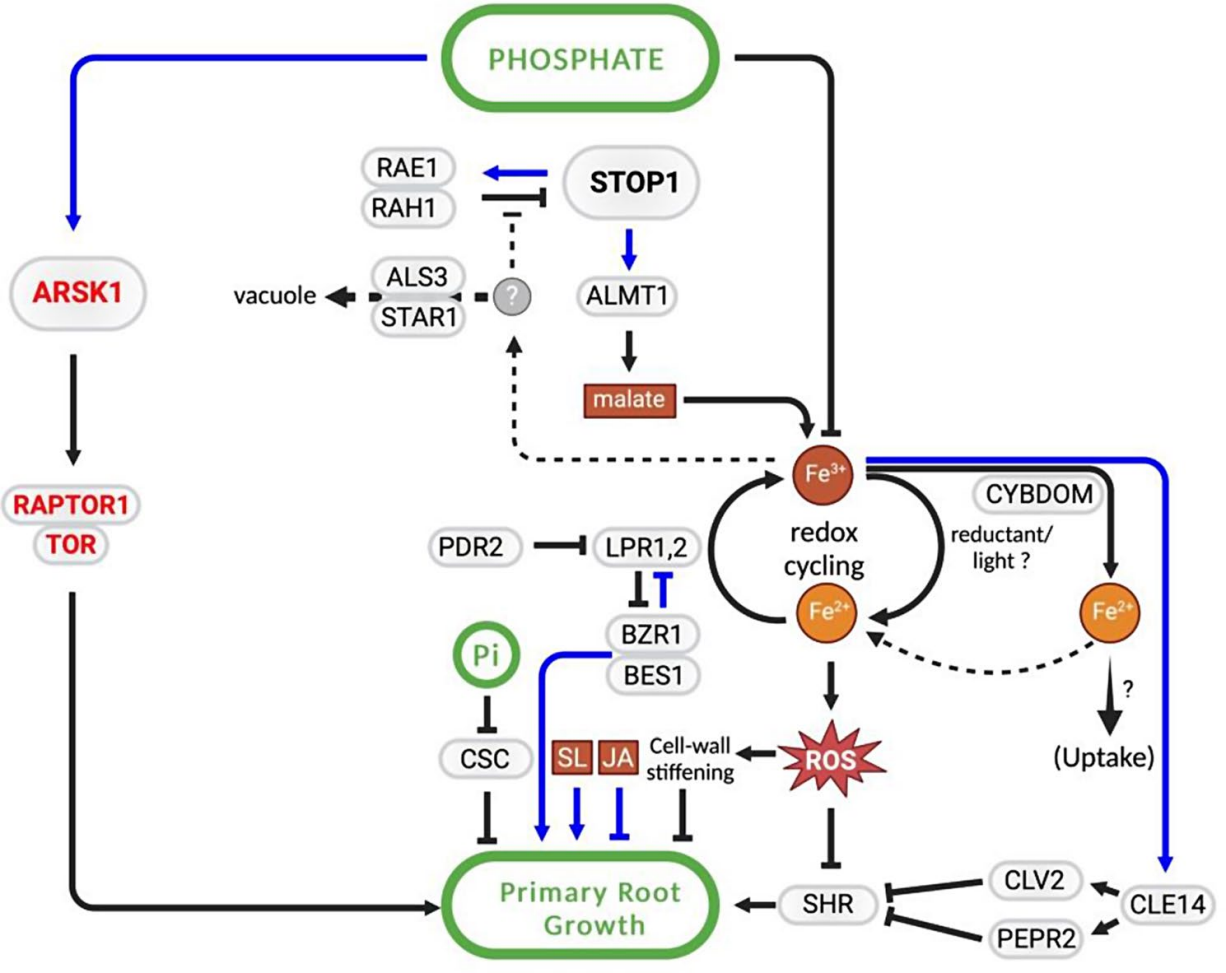
Schematic representation of local PS- and TORC1-signaling, controlling primary root growth. Genes are encircled in grey boxes. Metabolites, ions, reactive Oxygen species (ROS), and jasmonic acid (JA) and strigolactone (SL) phytohormones are encircled in red boxes. Components of TORC1 signaling and local PS signaling are represented in red and black letters, respectively. Cellulose synthase complex is denoted by CSC. Effects at the transcriptional and posttranscriptional levels are indicated by blue and black arrows, respectively. For details, see text

In Arabidopsis, the most extensively studied pathway for inhibiting primary root growth involves local PS signaling, which induces alterations in root system architecture and revolves around STOP1 TF and LPR1-like ferroxidases (for reviews, Abel 2017; Gutiérrez-Alanís et al. 2018). Briefly, in the presence of Fe, low Pi and acidic pH -which improves Fe solubility- in the medium induce increased STOP1 accumulation, leading to upregulation of the malate transporter gene and subsequent malate exudation (Balzergue et al. 2017; Godon et al. 2019; Mora-Macias et al. 2017). Malate, along with LPR1 and LPR2 ferroxidases, triggers iron redistribution in the apoplast of meristem and elongation zone cells (Balzergue et al. 2017; Mora-Macias et al. 2017; Svistoonof 2007). This redistribution induces the expression in the root apical meristem of *CLE14*, encoding a signaling peptide which acting through its CLV2 and PEPR2 receptors leads to meristem exhaustion (Gutiérrez-Alanís et al. 2017). Furthermore, LPR1 and LPR2 ferroxidases also contribute to a Fe redox cycle that induces reactive oxygen species (ROS) formation, resulting in callose deposition in the root apical meristem which impairs intercellular symplastic movement, leading to reduced accumulation of mobile SHORT ROOT (SHR), an important regulator of meristem maintenance, in the quiescent center (Müller et al. 2015). In addition, ROS prompts cell wall stiffening in the elongation zone, thereby inhibiting primary root growth (Balzergue et al. 2017; Mora-Macias et al. 2017; Fig. 1).

Recently members of the cytochrome b561 and DOMON domain (CYBDOM) protein family, named CRR and HYP1, were identified to exhibit ascorbate dependent iron reductase activity, which opposes the ferroxidase activity of LPR1,2, thereby modulating the ratio between Fe^2+^/Fe^3+^ in the root apoplast (Clua et al. 2024; Maniero et al. 2024). CRR and HYP1 presumably remove Fe^2+^ substrate for LPR1,2 ferroxidases by coupling with intracellular Fe^2+^ uptake, thereby attenuating LPR1,2-mediated ROS production and, concurrently, primary root growth inhibition (Clua et al. 2024; Maniero et al. 2024; Fig. 1). Consistent with this, mutation and overexpresion of these *CYBDOM* genes result in increased and decreased LPR1,2 mediated primary root growth inhibition, as well as shoot Fe accumulation, respectively (Clua et al. 2024; Maniero et al. 2024).

Other components in this pathway are PDR2, an endoplasmic reticulum (ER) localized P_5_-type ATPase that restricts the site of action of LPR1 and LPR2; RAE1 and RAH1 F-box proteins that mediate proteosomal degradation of STOP1; and ALS3/STAR1 that form a putative ATP-binding cassette transporter complex positively affecting RAE1 and RAH1 activities (Dong et al. 2017; Fang et al. 2021; Godon et al. 2019; Ticconi et al. 2009; Zhang et al. 2019; Fig. 1). Nonetheless, there remain gaps in our understanding of how low phosphate (Pi) triggers *STOP1* activation. The striking similarities between low Pi- and aluminum-triggered mechanisms regulating *STOP1*, such as their reliance on acidic pH and the modulation of *STOP1* activity at the translational level, suggest a shared pathway. In this regard, the aluminum receptor and the components of the pathway facilitating the positive regulation of *STOP1* by aluminum, involving ROS formation and the oxidation of RAE1, have been identified (Ding et al. 2024). However, the aluminum receptor, ALR1, a leucine-rich repeat (LRR) receptor-like kinase, is specific to aluminum and does not impact low Pi responses (Ding et al. 2024). Whether under low Pi conditions iron is perceived by a receptor related to ALR1 and subsequent signaling steps are shared with Al would be merit investigation.

Another pertinent aspect of local PS signaling, is the presence of a negative feedback loop between brassinosteroid (BR) signaling and LPR1. In this loop, LPR1 promotes increased translation of BKI, an inhibitor of BR receptors, thus preventing inhibition of BIN2 kinase that phosphorylates and inhibits BZR1 and BES1, closely related TFs that regulate BR responses and repress *LPR1* expression (Singh et al. 2018; Fig. 1).

A caveat regarding the biological significance of this local PS signaling pathway was noted by Zheng et al. (2019), who showed that low Pi triggered primary root growth inhibition requires direct root exposure to blue light, suggesting a role for a malate-mediated photo-Fenton reaction and a canonical Fenton reaction that form a Fe redox cycle in the root apoplast. However, it was argued that seeds germinate at the soil surface where roots can receive light (Raya-González et al. 2021). Moreover, Naumann et al. (2022) recently showed that low Pi triggered primary root growth inhibition occurs not only in light exposed roots, but to a lower extent it also occurs when roots are grown in dark conditions, affirming the operability of local PS signaling under natural growth conditions.

An additional mechanism contributing to root growth inhibition involves cellulose synthesis. Khan et al. (2024) demonstrated that PS stimulates cellulose synthesis, inducing alterations in cell wall thickness and structure, leading to primary root growth inhibition (Fig. 1). Consistent with these findings, the authors observed an elevation in cellulose synthase activity at the plasma membrane under PS conditions, likely stemming from reduced phosphorylation of cellulose synthase (Khan et al. 2024).

The study of Naumann et al. (2022) also provided strong evidence for the notion that land plant *LPR1,2* were acquired from soil bacteria via horizontal gene transfer, hypothesizing its instrumental role in the evolution of local PS sensing and PS adaptation during plant terrestrialization. Additionally, a role of LPR1,2 in iron transport and homeostasis beyond local PS sensing was recently described, and the same is likely to be the case for CYBDOM proteins (Clua et al. 2024; Xu et al. 2022).

While components of local PS sensing are conserved between Arabidopsis and rice, in rice, Pi starvation increases primary root growth and reduces lateral root number, a response involving strigolactones (see section: “PS-hormone signaling cross talks”).

TARGET OF RAPAMYCIN (TOR) is a serine/threonine kinase evolutionarily conserved in all eukaryotes, playing a pivotal role in cell growth regulation in response to various nutritional, stress and hormonal cues. In plants, TOR functions within the TORC1 complex, comprising RAPTOR, acting as a scaffold protein that recruits substrates for the TOR kinase, and LST8, a protein that binds to the kinase domain of TOR, essential for the full catalytic activity of TORC1 (Aylett et al. 2016; Burkart and Brandizzi 2021; Schmelzle and Hall 2000; Shi et al. 2018). While the implication of TORC1 in Carbon, Nitrogen and Sulfur signaling has been documented (reviewed by Burkart and Brandizzi 2021), the TORC1/PS signaling intersection has attracted less attention. In fact in the past, it was only in Chlamydomonas where it was found a TORC1/P-signaling connection, whereby PSR1 TF (the PHR1 counterpart in this organism; Bajhaiya et al. 2016) transcriptionaly controls the activity of LST8, and consequently that of TORC1 (Couso et al. 2020). However, recently in their study of the mechanisms of root growth inhibition under low Pi growth conditions, Cho et al. (2023) uncovered a mechanism for rapid reduction in root growth under low Pi conditions involving TORC1 de-activation by ASRK1 kinase, which is independent of iron (Fig. 1). Transcriptomic analysis and construction of a regulatory network underlying early responses to low P, led the authors to the identification of ARSK1 as a target of twelve interconnected TFs, revealing its repression by low Pi. Mutation of *ARSK1* and *ARSK1* overexpression result in reduced and increased root growth, respectively, indicating its role in root growth control. ARSK1 was found to act via TORC1 by phosphorylating RAPTOR1B, with mutation of *RAPTOR1B* also resulting in increased root growth arrest under low Pi (Cho et al. 2023). These findings underscore the involvement of the ARSK1-TORC1 module in root growth control under low Pi conditions, operating independently of local PS signaling that is iron-dependent. The question remains whether ARSK1 is part of the systemically controlled PS signaling pathway or represents an PHR1-independent signaling pathway.

**Fig. 2:**
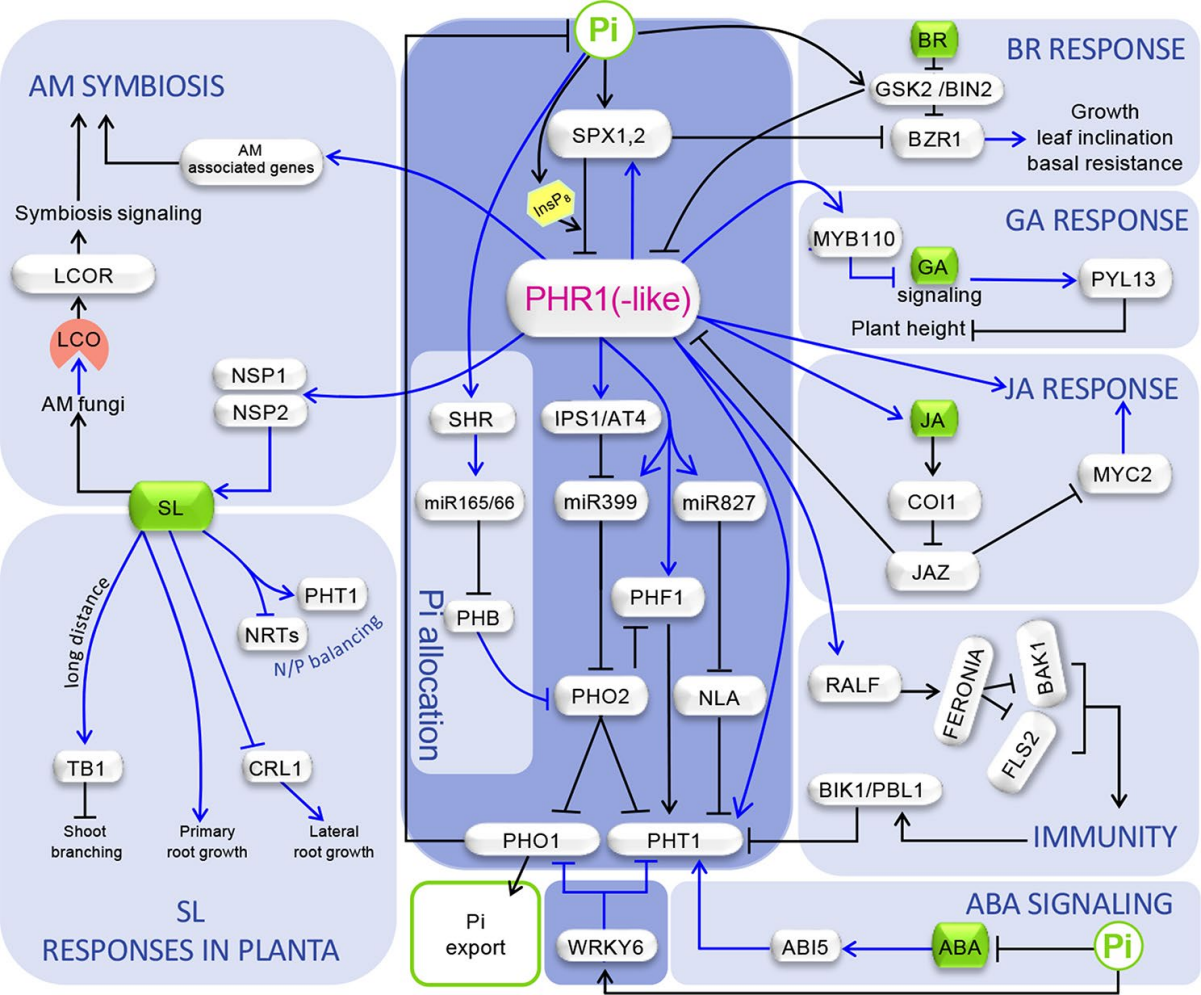
Schematic representation of regulatory effects and crosstalks associated with systemic PS signaling. The central panel containing previously known components of the core systemic PS signaling and the WRKY6 TF, controlling *PHO1* and *PHT1*, is shadowed in dark blue; other panels representing new information on the control of shoot Pi allocation involving a regulator of root development (SHR); the control of AM symbiosis; and crosstalks with hormones and immunity signaling are shadowed in light blue. Genes are encircled in grey boxes, and the master PHR1(-like) TFs are highlighted with red letters. Abscisic acid (ABA), strigolactores (SL), and jasmonic acid (JA) hormones, lipochitooligosaccharides (LCOs), and inositol pyrophosphate (InsP8) molecules are encircled in green, orange and yellow boxes respectively. LCO receptors, RLK10 in barley and NFR5 in rice, are denoted by a generic abbreviation (LCOR). Effects at the transcriptional and posttranscriptional levels are indicated by blue and black arrows, respectively. For details, see text

During Pi starvation, there is a significant reduction in shoot growth, partly mediated by the long-distance action of the phytohormone strigolactone (SL), which inhibits shoot branching (Kohlen et al. 2011; Umehara et al. 2008; Yuan et al. 2023; Fig. 2). Additionally, a new pathway of shoot growth inhibition in rice involving OsPHR2 control of OsMYB110 TF has been reported (Wang et al. 2024; Fig. 2). PS triggers OsPHR2-mediated induction of *OsMYB110*, negatively regulating plant height. Physiological and transcriptomic analyses revealed that *OsMYB110* does not affect PSR related to Pi acquisition and recycling and its effect on plant height primarily occurs via GA-signaling (Wang et al. 2024; Fig. 2). Thus, mutation of *OsMYB110* results in downregulation of repressors of gibberellic acid (GA) synthesis and signaling, and of GA oxidases, as well as in upregulation of *PIL13*, encoding a TF positively controlling internode elongation (Todaka et al. 2012). Interestingly, *Osmyb110* plants display higher grain yield both under high P and low P growth regimens, which is associated to increased grain number and weight per panicle (Wang et al. 2024). One striking finding has been that increasing and decreasing *OsMYB110* activity had a similar rather than opposite effect in lodging resistance (Wang et al. 2024). *OsMYB110* overexpressing plants increased lodging resistance is associated to their shorter size and increased lignin content, whereas in *Osmyb110* plants despite their higher height and lower lignin content, increased lodging resistance is postulated to be caused by the increased diameter and thickness of the internode in this mutant (Wang et al. 2024).

In addition to *OsMYB110* acting via GA signalling, the control of growth by PS signaling also involves the participation of BR and Jasmonic acid (JA) signaling (see section: “PS-hormone signaling cross talks”).

### Control of pi distribution in plants

Pi acquisition and distribution are controlled by the systemic PS signaling pathway, which is highly conserved in plants. This pathway revolves around two key players, PHR1 and its counterparts (PHR1-like; PHL) TFs of the MYB-CC family (containing a MYB DNA-binding domain an a coiled coil dimerization domain) and SPX1 and related proteins acting as sensors for insositol pyrophosphate InsP8, a Pi-rich compound whose levels correlate with those of Pi (Dong et al. 2019; Ojeda-Rivera et al. 2022; Paz-Ares et al. 2022; Puga et al. 2014; Rubio et al. 2021; Wang et al. 2014; Wild et al. 2016; Zhou et al. 2008; Zhu et al. 2019; Fig. 2). Essentially, in the presence of Pi, high InsP8 levels prompt SPX-binding and the concomitant inhibition of PHR1(-like) TFs (Dong et al. 2019; Zhu et al. 2019). Conversely, in the absence of Pi, the lack of InsP8 prevents SPX binding to PHR1, leading to the transcriptional program for Pi starvation. Structural-functional analyses involving rice SPX2, PHR2, and InsP6 (a commercially available substitute for InsP8) revealed that in the SPX2-InsP6-PHR2 complex, SPX2 assembles into a domain-swapped dimer form that binds two copies of PHR2 monomers through both their DNA binding and coiled-coil domains (in a 2:2 stoichiometry), thereby inhibiting DNA binding and disrupting PHR2 dimerization (Guan et al. 2022). Similarly, a comparable negative effect on PHR2 DNA binding and dimerization was observed for rice SPX1 in the structural-functional analysis conducted by Zhou et al. (2021), although in this instance, a SPX1 monomer binds to a PHR2 monomer (in a 1:1 stoichiometry).

Downstream targets of PHR1 include genes encoding high-affinity PHT1 Phosphate transporters, phosphatases, and RNases involved in Pi mobilization, acquisition, and recycling. Additional important regulators in this systemic signaling pathway include PHO2, a ubiquitin conjugating E2 enzyme, that prompts the degradation of Pi exporter PHO1, and together with NLA RING-type ubiquitin E3 ligase, mediates the degradation of PHT1 Pi transporters (Aung et al. 2006; Bari et al. 2006; Lin et al. 2013; Liu et al. 2012; Park et al. 2014). In addition, PHO2 controls the accumulation of PHF1, an ER protein that enables PHT1 exit from the this compartment to traffic to the plasmamembrane (Gonzalez et al. 2005; Huang et al. 2013). The control exerted by PHR1 over PHO2 and NLA is indirect and occurs via the upregulation of *miR399* and *miR827*, two miRNAs displaying shoot to root mobility, guiding *PHO2* and *NLA* mRNA degradation, respectively (in Arabidopsis; in rice miR827, does not target *NLA*; Bari et al. 2006; Chiou et al. 2006; Fujii et al. 2005; Lin et al. 2008; Pant et al. 2008). PHR1 also modulates *IPS1* and related non-coding RNAs that inhibit miR399 activity (Franco-Zorrilla et al. 2007). Additionally, the expression of *PHO1* and *PHT1* is downregulated by WRKY6, a TF degraded under low Pi conditions (Castrillo et al. 2013; Chen et al. 2009; Ye et al. 2018; Fig. 2).

A challenging aspect of this systemic PS signaling model concerns the control of root-to-shoot Pi translocation under low Pi growth conditions. Indeed, according to it, the more severe the Pi limitation, the more *PHO1* activity increases, which would lead to Pi exhaustion in the root by translocation to the shoot. However, this possibility contradicts the observed increase in the root-to-shoot growth ratio during Pi starvation. A recent study has shed light on this contradiction by revealing a new regulatory loop involving indirect positive control of *PHO1* by *SHR*, a key regulator of meristem maintenance as well as of radial patterning in the root (Helariutta et al. 2000; Xiao et al. 2022; Fig. 2). Through a direct genetic approach, these authors found that mutation of *SHR* resulted in reduced Pi accumulation in the shoot (Xiao et al. 2022). Further investigation uncovered the regulatory pathway explaining SHR’s effect on Pi translocation. Briefly, SHR upregulates miR156, which post-transcriptionally inhibits the activity of the *PHB*. In turn, PHB directly upregulates *PHO2*, leading to increased PHO1 degradation (Xiao et al. 2022). This regulatory loop balances root/shoot growth ratio with Pi status: when Pi is low, reduced *SHR* activity increases *PHB* and *PHO2* activity, enhancing PHO1 degradation and Pi retention in the root, thereby alleviating root growth inhibition. In summary, this regulatory loop exemplifies how integrating developmental and nutritional signaling pathways coordinates differential organ growth with nutritional status. It would be valuable to examine how Pi levels control SHR activity.

In recent years, various methods have emerged to analyze phosphate (Pi) translocation and distribution at the sub-organ, cellular, and/or subcellular levels, employing both physical and biological procedures to address the significant challenge of understanding Pi homeostasis in plants. Physical methods include ^31^P magnetic resonance spectroscopy, which enables quantification of Pi in different subcellular compartments but lacks cellular resolution, and techniques utilizing ^32^P or ^31^P radioisotopes, which allow analysis of kinetic parameters of Pi uptake and imaging Pi distribution using high-resolution live radioisotope micro-imaging systems, that reaches up to100 µm resolution (Kanno et al. 2012, 2016a, b). Biological methods rely on the use of genetically-encoded Fluorescence Resonance Energy Transfer (FRET) sensors for phosphate (FLIPPi) (Assunção et al. 2020; Gu et al. 2006; Mukherjee et al. 2015; Sahu et al. 2020; for a recent review see, Sadoine et al. 2023). While these methods provide valuable insights on cellular and subcellular distribution of Pi, they are time-consuming and impractical for many applications due to the requirement for specialized equipment and transgenic introduction of sensor constructs. A recent technical breakthrough by Guo et al. (2024) enables easy in situ histochemical visualization of intracellular Pi at the cell resolution level. This semiquantitative high-resolution technique has revealed profound intercellular differences in intracellular Pi content in Arabidopsis and rice, as well as the impact of mutations at key Pi signaling components in Pi distribution. Furthermore, this technique shows promise for screening mutants with altered cellular distribution of Pi and for natural variation studies of intracellular Pi distribution. For instance, Guo et al. (2024) identified a *pho1;1* mutant in rice displaying higher Pi accumulation in leaf tips. They propose that PHO1;1 Pi exporter activity in companion cells and xylem parenchyma cells prevents Pi overaccumulation in leaf tips. Additionally, mutation of *PHO1;1* and its close paralog *PHO1;3* also results in increased Pi accumulation in the husk’s spongy parenchymal cells and the developing seed’s nucellar epidermal cells. These findings are in line with those of another study by Ma et al. (2021), who uncovered a role for *OsPHO1;2* in preventing Pi overaccumulation in the rice endosperm which would inhibit starch biosynthesis enzymes and grain filling.

### PS impact on plant-microbe interactions

Over the past five years, numerous studies have underscored the significance of Pi limitation in plant-microbe interactions, elucidating the involvement of PS signaling in this phenomenon (for recent reviews, see Paries and Gutjahr 2023; Zhao et al. 2023). Concerning P nutrition, the most emblematic plant-microbe association is with arbuscular mycorrhizal (AM) fungi, a symbiotic relationship prevalent across the plant kingdom (observed in over 80% of plant species). It has long been recognized that low Pi levels promote AM symbiosis. Early research highlighted the role of plant-derived flavonoids and SL phytohormones as signals initiating AM symbiosis (Akiyama et al. 2002, 2005; Nair et al. 1991; Paries and Gutjahr 2023). Recent investigations have provided further insights into the pivotal roles of SLs and SL-related molecules, such as Karrikin-like compounds, in triggering AM symbiosis, via the common symbiosis signaling pathway, which is also involved in the establishment of the legume-rhizobia symbiosis (Choi et al. 2020; Li et al. 2022; Parniske 2008; Fig. 2). PS induces the synthesis of SLs and Karrikin-like molecules through the upregulation of biosynthesis genes by NSP1 and NSP2 TFs. These compounds are then exuded, promoting hyphal branching of mycorrhizal fungi and the release of fungal signals, like lipochitooligosaccharides (LCOs), which are recognized by plant LCO receptors, such as RLK10 in barley and NFR5 in rice, thereby activating common symbiosis signaling (Li et al. 2022). Notably, the upregulation of *RLK10/NFR5* is mediated by Karrikin-like molecules, which promote the degradation of the SMAX1 repressor controlling *RLK10/NFR5* expression (Choi et al. 2020). The upregulation of *NSP1* and/or *NSP2* by PHR1 already informs on the important role of systemic signaling in the control of AM symbiosis. This PHR1-centric scenario has been expanded with recent studies revealing a more direct and broader implication of PHR1 and SPX sensors in AM establishment and functioning (Fig. 2). Thus, in a key study to dissect the plant regulatory network underlying AM symbiosis in rice, Shi et al. (2021) performed a yeast one hybrid screen of 1570 TFs with the promoters of 51 rice genes associated with AM symbiosis. These authors found that PHR2 was placed as a central hub in the regulatory network underlying symbiosis. In line with this, inactivation of *PHR2* and its close paralogs severely impairs AM colonization and development, while overexpression of *PHR2* has the opposite effect (Shi et al. 2021). Das et al. (2022) independently reported the key role of *PHR*2 in AM symbiosis, demonstrating that many AM-associated genes are direct targets of PHR2, including genes of the common symbiosis signaling, and paralleling the results of Shi et al. (2021), a *phr2* mutant displayed reduced root colonization, mycorrhizal Pi uptake, and crop yield. This finding was extended to tomato by Liao et al. (2022). In agreement with the inhibitory activity of SPX1 and related sensors on PHR1 activity, studies by Shi et al. (2021) and Liao et al. (2022) also showed that inactivation of SPX1-related functions resulted in increased AM colonization in rice and tomato. However, a paradoxical situation arises in *Medicago truncatula*, where inactivation of SPX1 and SPX3, two highly related homologs of Arabidopsis and rice SPX1, results in reduced AM colonization, despite their interaction with PHR1 (Wang et al. 2021). Nevertheless, *spx1spx3* mutant plants display an increase in large arbusculatures, accompanied by the downregulation of arbusculature degeneration-associated genes, suggesting a role for these proteins in arbuscular degeneration (Wang et al. 2021). The negative impact of *spx1spx3* double mutation on AM colonization could partly be attributed to the reduction in SL levels associated with these mutations (Wang et al. 2021). Additionally, the phenotypic consequences of *spx1spx3* double mutation may reflect a non-canonical effect of SPX1 and SPX3 via a regulator other than PHR1. Indeed, the possibility that SPX1 action extends beyond PHR1-like TFs has been documented. Specifically, in soybean, GmSPX5 exerts its action via interaction with GmNF-YC4, that increases the DNA-binding affinity of this TF, leading to increased nodule number and fresh weight (Zhuang et al. 2021).

The non-canonical mechanism of action of GmSPX5 is associated to a non-typical function of this protein class, not directly related to Pi homeostasis, but rather it appears associated to nodule development (Wang et al. 2021). In any case, nodule function depends on a proper supply of Pi to the bacteria (Tang et al. 2001), and in this context the importance of the PHR1-PHT1 module in nodule development under Pi limitation has also been substantiated (Chen et al. 2019; Lu et al. 2020). Thus, overexpression of *GmPHR1* enhances the expression of the *PHT1* transporter gene in nodules, resulting in increased Pi content and nodule size (Lu et al. 2020).

Beyond its impact on AM symbiosis and nodulation, Pi levels have been shown to affect the root-associated microbiome (Castrillo et al. 2017; Fabiańska et al. 2019; Finkel et al. 2019; Yu et al. 2018). In the study by Castrillo et al. (2017) it was found that under low Pi growth conditions, a synthetic bacterial immunity (SYNCOM) reduced shoot Pi and growth, indicating bacterial competition with plants for Pi. Subsequent transcriptomic analysis of wild-type and *phr1(-like)* mutants revealed that PHR1 represses plant defenses against pathogens, a finding validated by enhanced resistance to *Pseudomonas syringae* and the oomycete *Hyaloperonospora arabidopsidis* in *phr1(-like)* mutants (Castrillo et al. 2017). Tang et al. (2022) recently elucidated a mechanism explaining the reduced pathogen defense of PHR1 under low Pi, by showing that PHR1 directly targets and upregulates genes encoding RAPID ALKALINIZATION FACTORs (RALFs; Fig. 2). These proteins inhibit plant defense by interacting with FER kinase, thereby inhibiting the interaction between FLS2 and BAK1 kinases (Tang et al. 2022). While relieving plant defenses under low Pi conditions could lead to the colonization by latent opportunistic bacterial competitors, it has also been shown to promote the recruitment of beneficial bacteria (Finkel et al. 2019; Tang et al. 2022). Thus, it appears plausible that plants reduce their defense systems to facilitate interactions with beneficial microorganisms, although this is associated with the potential trade-off of enabling the establishment of opportunistic microorganisms.

Mirroring the effect of PHR1 on reducing plant defenses, it has also been reported that the plant immunity signaling system negatively affects Pi uptake. Dindas et al. (2022) found that upon elicitation, the receptor-like kinases BIK1 and PBL1 phosphorylate the PHT1;4 transporter in Arabidopsis, causing its inactivation and enhancing immunity (Fig. 2). Altogether, there is a complex interplay between immunity and PS signaling governing the outcome of plant-microbe interactions under low Pi growth conditions.

### PS-hormone signaling crosstalks

For many years, extensive crosstalk has been observed between PS signaling and the signaling pathways of all major classes of phytohormones, including abscisic acid (ABA), auxin, brassinosteroids (BR), cytokinins, ethylene, gibberellin (GA), jasmonic acid (JA), and strigolactones (SL; for reviews, see Paz-Ares et al. 2022; Rubio et al. 2009; Scheible and Rojas-Triana 2015). In addition, Pi starvation signaling displays a wide array of crosstalks with the signalling pathways of several nutrients, such as N, Fe and Zn as well as with that of its chemically similar toxic compound, arsenate (reviewed by Paz-Ares et al. 2022). Here we focus on recent advances that have shed new light on the mechanisms underlying the crosstalk with SLs, BR, JA, and ABA.

SL serve several functions during Pi starvation, in addition to acting as secreted signals to promote AM symbiosis mentioned above. They are known to promote the elongation of the primary root and the reduction of lateral root numbers (Sun et al. 2014), and SLs also act as long-distance signals within plants to repress shoot branching via activation of TEOSINTE BRANCHED1 (TB1) TF (Wang et al. 2018, 2020). Recently, Yuan et al. (2023) carried out a detailed examination the PS: SL signaling crosstalk in rice. In addition to confirming the importance of *PHR2* controlled *NSP1* and *NSP2* on induction of SL biosynthesis genes during Pi starvation, they showed the role of SLs in reducing lateral root density under low Pi conditions in rice involve repression of *CROWN ROOTLESS1* (*CRL1;* Fig. 2). Importantly, Yuan et al. (2023) also uncovered that SLs trigger the upregulation of *PHT1* transporter genes while they downregulate the expression of genes for nitrate and ammonium transporters, thereby adding a new mechanism to balance Pi and nitrogen acquisition. Furthermore, the potential of *NSP1* and *NSP2* as tools for Pi acquisition and use efficiency (PUE) improvement has been found by showing that a moderate increase in the expression of *NSP1* and *NSP2* in transgenic plants harboring extra doses of these genes, results in improved Pi uptake and grain yield under low and moderate Pi growth conditions (Yuan et al. 2023).

In terms of BR, it was previously known that low Pi reduces the activity of BZR1 and BES1 TFs involved in BR responses through a LPR1-dependent posttranslational mechanism, which regulates shallow root architecture (Singh et al. 2018; Fig. 1). Besides this crosstalk between BR and local PS signaling, another interplay between BR and systemic PS signaling has been identified in rice, involving the interaction between SPX1,2 and BZR1 (He et al. 2024; Fig. 2). Interestingly, contrary to the InsP8 dependence of the SPX1,2-PHR1 interaction, the SPX1,2-BZR1 interaction is independent of InsP8, thus enabling repression of BZR1 activity under low Pi conditions when InsP8 levels are low but those of SPX1,2 are high (He et al. 2024). This crosstalk leads to inhibition of BR responses, such as growth and leaf inclination, under low Pi (He et al. 2024). Additionally, BZR1 positively regulates the biosynthesis of flavonoids, particularly sakuranetin, a phytoalexin effective against some fungi such as *Magnaporthe oryzae*. It is hypothesized that BZR1 control of sakuranetin contributes to the maintenance of basal resistance (He et al. 2024). The reduction of BZR1 basal resistance under PS is compensated by PS activation of JA signaling which leads to overaccumulation of sakuranetin (He et al. 2024).

Another crosstalk between Pi starvation and BR signaling is mediated by RLI1, a PHR1 homolog that undergoes alternative splicing (AS) producing two transcripts encoding RLI1a and RLI1b proteins (Guo et al. 2022a; Ruan et al. 2018). RIL1b proteins contain both a MYB and a coiled-coil (CC) domain and, similar to PHR1, control the expression of Pi starvation response-related genes. RLI1a lacks the CC domain and has a broader range of targets, including most RLI1b targets as well as other targets such as BR biosynthesis (such as *D11*, *DWF4*, and *CYPD903*) and BR signaling genes (such as *GSK3* and *BZR1*). *RLI1a* mRNA and protein accumulation are reduced by Pi starvation, while *RLI1b* transcript is nonresponsive, whereas RLI1b protein accumulation is increased by Pi starvation (Guo et al. 2022a).

As a result of these two crosstalks, BR-controlled processes such as leaf inclination, which enhances light capture and increases photosynthetic activity, and growth, both of which require Pi, are inhibited, thereby reducing Pi needs. Additionally, a recent discovery has unveiled a new BR/PS signaling crosstalk mechanism in rice. In this mechanism, the BR-signaling inhibitor GSK2, a functional homolog of BIN2 kinase, phosphorylates PHR2 at serine 239 residue, impairing its DNA binding activity (Zhang et al. 2024; Fig. 2). Conversely, these authors found that, like BRs, low Pi triggers GSK2 destabilization (Zhang et al. 2024). Consequently, low Pi relieves GSK2 inhibition of PHR2. This crosstalk mechanism could help adjust PS responses to the Pi acquisition demand of BR-controlled processes. Thus, when BR levels are low and Pi levels are high, the demand for Pi acquisition is low, and GSK2 inhibits PHR2. Conversely, when BR levels are high or Pi levels are low, increasing PHR2 activity (through GSK2 destabilization) is necessary to satisfy the Pi demand.

In the context of JA signaling, previous studies indicated that PS triggers enhanced JA accumulation, partly dependent on PHR1, leading to increased herbivory resistance and growth inhibition (Khan et al. 2016). MYC2 and related TFs, JAZ repressors, and the jasmonate receptor COI1 are key players in JA signaling (Fonseca et al. 2009). He et al. (2023) elucidated the crosstalk mechanism between JA and PS signaling, revealing that MYC2 interacts with PHR1(-like) to synergistically activate JA-responsive genes, including JA and sakuranetin biosynthesis genes (He et al. 2023, 2024; Fig. 2). Moreover, several JAZ proteins also interact with PHR1(-like) TFs, and overexpression of JAZ leads to reduced Pi starvation/JA responses (He et al. 2023). Therefore, there is a tight positive bidirectional interplay between Pi starvation- and JA-signaling which underscores the importance of balancing P homeostasis with growth and defense.

While the role of ABA in plant responses to various abiotic stresses is well established (Zhu 2016), its implication in controlling PSR has been less understood. Zhang et al. (2022) provided new insights into the ABA: PS signaling connection, showing that PS results in increased ABA levels (Fig. 2). Impairment of ABA biosynthesis leads to reduced expression of *PHT1;1* and *PHT1;4*, resulting in decreased Pi content and shorter roots under low Pi conditions. Furthermore, ABI5, an ABA signaling TF induced by Pi starvation, acts as a direct regulator of *PHT1;1* expression and positively controls root growth under low Pi conditions (Zhang et al. 2022). Interestingly, the upregulation of *ABI5* by PS is independent of PHR1 (Zhang et al. 2022), indicating cross talk between PS signaling and ABA that is at least partially independent of canonical long-distance systemic signaling.

### A new regulatory mechanism of microRNA activity by an antisense RNA in PS signaling

The study of PS signaling has significantly contributed to our understanding of miRNA and long non-coding RNA (lncRNA) biology (reviewed by Paz-Ares et al. 2022). Recently, Wang et al. (2023) revealed a novel regulatory mechanism of microRNA activity in maize, where a maize natural antisense RNA protects the mRNA of *PHT1*gene*s* from miR399-guided degradation. Indeed, these authors found that several maize *PHT1* family members contain target sites for miR399, and they demonstrated the functionality of these sites for three *PHT1* mRNAs (*ZmPHT1;1, ZmPHT1;3* and *ZmPHT1;13*). Furthermore, a *cis*-natural antisense RNA, *PILNCR2*, is transcribed from the complementary strand of *ZmPHT1;1* (Wang et al. 2023). Subsequently, these authors showed that *PLNCR2* and *PHT1* RNAs could form heteroduplexes in vivo and in vitro, and that overexpression and mutation of *PILNCR2* result in increased and decreased *PHT1;3* and *PHT1;13* mRNA levels, respectively. Consistent with these molecular effects, increasing and decreasing *PLNCR2* enhances and diminishes low Pi tolerance in maize. Overall, these findings indicate that heteroduplex formation with *PILNCR2* prevents the targeting of *ZmPHT1* RNAs by miR399. It will be intriguing to investigate the prevalence of this regulatory mechanism in other plants and eukaryotic organisms in the context of PS and beyond.

### Advances in natural variation studies of the PSR

Natural variation, has been the fuel of breeding efforts since the beginning of agriculture as it provided the genetic diversity from which to select optimal allelic combinations. The development of tools for high-throughput genotyping, and methods for genetic analysis and mapping of natural variation has contributed to improving the efficacy of breeding programs, and also made natural variation studies gain the momentum towards the goal of understanding the molecular basis of adaptation to local environments as well as towards the discovery of new genes affecting virtually any phenotypic trait (Alonso Blanco et al. 20009). While numerous studies have identified Quantitative Trait Loci (QTL) related to phosphate (Pi) acquisition and use efficiency (PUE), only a few have successfully pinpointed the causal genes underlying these QTLs (Ojeda-Rivera et al. 2022; Paz-Ares et al. 2022). Notable examples of identified causal genes include *PHOSPHORUS STARVATION TOLERANCE1*, which encodes a kinase enhancing early root growth that results in increased soil exploration and Pi acquisition capacity in a PSR independent manner (Gamuyao et al. 2012), and *LPR1*, encoding a ferroxidase involved in local PS signaling, as discussed above (Svistoonoff et al. 2007).

Recent genome-wide association studies (GWAS) in Arabidopsis have revealed genetic diversity at a cluster of *PHT1* transporter genes responsible for natural variation in Pi accumulation/uptake (Chien et al. 2022; Yi et al. 2021). Notably, Chien et al. (2022) found a significant association between low and high Pi uptake *PHT1* haplotypes and topsoil Pi content, suggesting *PHT1s* could be potential targets for adaptation to habitats with different Pi levels. In addition, Yi et al. (2021) identified *PILS7*, encoding an auxin efflux carrier protein, as a causal gene underlying a QTL for Pi uptake and root and shoot growth under high or low Pi conditions, further underlining the known impact of auxin signaling on PSR (Bhosale et al. 2018; Huang et al. 2018). Furthermore, in the GWA study of Chien et al. (2022), seven additional genes, whose products among others include protein phosphatases, casein kinases, and MYB52 TF, were shown to affect plant Pi accumulation. Additionally, the authors showed that MYB52 acted as a new regulator of *PHT1* expression.

Another GWA study has uncovered the impact on Pi uptake of AtPITP7, a chloroplast Sect. 14-like protein in Arabidopsis (Yang et al. 2023). Further analyses of lipid composition and transcriptome of overexpressors and/or mutants pointed to the participation of Sec 14-like protein in the regulation of the prokaryotic lipid biosynthesis pathway leading to the production of sulfolipids (Yang et al. 2023). Additional GWA studies in Arabidopsis have led to the identification of *LYSO-PHOSPHATIDYLCHOLINE ACYLTRANSFERASE 1*, relevant in the modulation of Pi content by Zn (Kisko et al. 2018); *VARIANT IN METHYLATION, FORMIN-LIKE PROTEIN 6, and VOLTAGE-DEPENDENT ANION-SELECTIVE CHANNEL PROTEIN 3* that control root growth under combined iron-phosphorus deficiency (Bouain et al. 2019); and *PHT4;4*, encoding a chloroplastic ascorbate transporter, and *bZIP58*, which prevent downregulation of photosynthesis genes caused by combined iron-phosphorus deficiency (Nam et al. 2021). Also, following a GWA approach in *Lotus japonicus*, an LRR receptor and a cytochrome B5 reductase were found to influence Pi accumulation in plants grown under sufficient Pi conditions (Giovannetti et al. 2019). A particularly interesting finding stems from a GWAS study in soybean, where a robust signal associated with phosphate (Pi) acquisition was identified at a locus named *CPU1 (COMPONENT OF PHOSPHORUS UPTAKE 1)*, and the causal gene underlying this effect was identified as *GmPHF1*, the functional homolog of Arabidopsis *PHF1*, which facilitates PHT1 transporter trafficking to the plasma membrane (Guo et al. 2022b). Detailed functional analysis revealed that a single nucleotide polymorphism (SNP) within an upstream open reading frame (uORF) leads to altered abundance of GmPHF1 in a tissue-specific manner, consequently resulting in changes in Pi acquisition (Guo et al. 2022b).

In the majority of these examples of successful identification of genes impacting PSR-related traits, selection of candidates made use of available information on gene characteristics (e.g., PS responsiveness, gene function) or even on gene co-function networks as well as on haplotypes. Efforts to generate multiomics datasets in the collections of accessions grown under suitable Pi regimens, that would empower natural variation studies, are still at an incipient stage. In fact, so far, there have only been reported metabolomics and/or transcriptomic and ionomic studies focused on selected subsets of accessions/cultivars displaying high or low tolerance to limiting Pi growth conditions. In general, these studies identified some significant correlations between gene co-expression modules, metabolites, and/or elements with plant performance traits under low Pi growth conditions, which inform on metabolic pathways and transcriptional programs and potential TF regulators underlying low Pi tolerance, functional validation is still missing for most candidates (Hajheidari et al. 2022; Han et al. 2022; He et al. 2022; Luo et al. 2019). Only in one GWAS study in maize, the information on Pi starvation-responsive metabolites and the significant correlation between specific metabolites and plant performance traits has been used to identify potential candidate causal genes underlying QTLs for these traits. One of these candidates was a gene encoding a glucose-6-phosphate-1-epimerase that was preliminarily validated as a causal gene of a QTL for yield in a recombinant inbred population (Luo et al. 2019).

An additional type of natural variation study has focused on specific PS-related genes identified through reverse genetics approaches to examine their contribution to the natural variation of PS-related traits. Thus, in Arabidopsis *PHO1*, it was found that expression of a mutant that impairs an uORF results in enhanced *PHO1* translation and increased Pi content, improving shoot growth under low Pi conditions. An examination of sequenced Arabidopsis accessions showed that 1% of them has mutations impairing the uORF, and these accessions display higher shoot Pi accumulation than the reference Col accession that has a functional uORF. These findings highlight the potential of selecting *PHO1* variants with impaired uORF (or of generating them by gene editing) in breeding programs for PUE (Reis et al. 2020) and, together with the findings by Guo et al. (2022b) in *GmPHF1*, underline the potential of mutations targeting uORFs that alter translation to impact natural variation of adaptive and/or agronomic traits. Likewise, in their study of *Osmyb110*, involved in the control of plant height, Wang et al. (2024) found that genetic diversity at this gene is associated with alteration in plant height and growth performance both in indica and japonica cultivars, with haplotype 2 (hap2) accessions having lower levels of *OsMYB110* mRNA and displaying higher plant height, placing this haplotype (or of an equivalent gene-edited variant) as a prime candidate for breeding (Wang et al. 2024). Additionally, in an independent study in soybean, Wang et al. (2022) found that overexpression of *GmPHR14* and *GmPHR32* in soybean hairy roots significantly increased root Pi content and root and shoot biomass under low Pi growth conditions. An examination of genetic diversity in *GmPHR14* and *GmPHR32* identified three haplotypes for each gene in a collection of soybean accessions, where haplotype 2 of both genes was associated with higher shoot dry weight under low P growth conditions. Moreover, the authors examined the impact of domestication and breeding on haplotype selection and found that for *GmPHR14*, hap2 representation was 0%, 6%, and 25% in wild accessions, landraces, and improved varieties, respectively, while for *GmPHR32*, hap2 representation in these accessions classes was 8%, 33.5%, and 51%. These findings prioritize the haplotype 2 for these genes as candidates for positive selection during domestication and breeding, and warrant their follow-up in future breeding programs for PUE improvement (Wang et al. 2022).

## Conclusions and perspectives

Intensive effort has been made in the study of the PSR and its regulation since the beginning of this century, which has led to the identification of many components of this pathway and the interactions among themselves as well as with components of signaling pathways of other nutrients, hormones, developmental processes and stress responses. The trend in the effort has not changed in the near past as demonstrated by the number of key advances that occurred recently in the field.

As examples, these recent advances have disclosed the role of TOR signaling in the control of root growth under PS and the integration of a (SHR-associated) root developmental pathway in the control of shoot-root Pi allocation. Moreover, the development of simple methodology to examine intracellular Pi distribution opens new opportunities to identify genes and signals determining Pi distribution in planta and its dynamics, by large scale genetic screens as well by analysis of P distribution in collections of accessions/cultivars of different plant species. Also noteworthy are the newly disclosed crosstalks between hormones and PS signaling that influence growth and developmental pathways, and the bidirectional negative interplays between PS and immunity signaling. Recent findings showing the key role of Pi systemic signaling in the control of AM symbiosis, as well as on the establishment of the microbiome under low Pi growth conditions open new opportunities to improve PUE by manipulating AM-symbiosis/microbiome assembly. Certainly, the complexity of (re-)assembling microbiome communities in response to alterations in Pi availability still requires a great effort to unravel the underlying mechanisms and candidate (rational-)breeding targets.

Another important recent discovery has been the mechanism of inhibition of miRNA activity based on protection of the target by heteroduplex formation with an antisense RNA. Whether this mechanism is specific for the control of miR399 or is extended to other miRNAs in plants and other organisms, as it is the case of target mimicry, will delimitate the importance of this regulatory strategy in miRNA biology. Finally, natural variation studies are still at their infancy, but the cases reported already show the power of this approach to discover new genes and processes involved in adaptation of plants to habitats with different levels of available Pi, and in the identification of targets for breeding of PUE traits. These results grant the implementation in the near future of ambitious natural variation programs involving the generation of multiomics datasets in the collections of accessions from different plant species grown under suitable Pi regimens which will allow the implementation of effective (comparative-) systems biology approaches.

Altogether, the knowledge gathered from the studies of the PSR certainly satisfy the most ambitious expectations from the basic research perspective, as we have gained enormous insights on the problem of Pi homeostasis from an integrated metabolic, nutritional, developmental and plant microbe interaction perspective, which highlights the hierarchically high position of PS signaling and P-homeostasis in plant physiology and development. Moreover, these studies have also led to the identification of new regulatory mechanisms of miRNA activity whose implications already extend well beyond the field of Plant Biology, qualifying the plant PSR as a model system beyond rhetoric. From the applied perspective towards the goal of obtaining plants with improved PUE, while logically advances lag behind basic discoveries, there are already some results that hint on the potential applications of some of the genes disclosed in the study of the PSR, such as for instance *PHR1(-like), PHO1, PHF1, MYB110, NSP1/NSP2* etc. The future is promising….
